# Machine learning to predict ceftriaxone resistance using single nucleotide polymorphisms within a global database of *Neisseria gonorrhoeae* genomes

**DOI:** 10.1128/spectrum.01703-23

**Published:** 2023-10-31

**Authors:** Sung Min Ha, Eric Y. Lin, Jeffrey D. Klausner, Paul C. Adamson

**Affiliations:** 1 Department of Integrative Biology and Physiology, UCLA, Los Angeles, California, USA; 2 David Geffen School of Medicine at UCLA, Los Angeles, California, USA; 3 Departments of Population and Public Health Sciences and Medicine, Keck School of Medicine of University of Southern California, Los Angeles, California, USA; 4 Division of Infectious Diseases, David Geffen School of Medicine at UCLA, Los Angeles, California, USA; University at Albany, Albany, New York, USA

**Keywords:** machine learning, *Neisseria gonorrhoeae*, ceftriaxone, antibiotics, antimicrobial resistance

## Abstract

Antimicrobial resistance (AMR) in *Neisseria gonorrhoeae* is an urgent global health issue. Machine learning (ML) is a powerful tool that can aid in identifying mutations and predicting their impact on AMR. The study aimed to use ML models to predict ceftriaxone susceptibility and decreased susceptibility (S/DS). A public database of *N. gonorrhoeae* genomes with minimum inhibitory concentration (MIC) data was used to evaluate seven ML models using 97 single nucleotide polymorphisms (SNPs) known to be associated with ceftriaxone resistance. Ceftriaxone MICs ≤ 0.064 mg/L were classified as susceptible, and ceftriaxoneMICs > 0.064 mg/L were classified as DS. The contributions of individual SNPs to predict S/DS were calculated using SHapley Additive exPlanation (SHAP) values. An ML model was retrained using different combinations of SNPs with the highest SHAP values. The performance of ML models was assessed using different metrics including area under the curve (AUC) and balanced accuracy (bAcc). The ML analyses included 9,540 *N*. *gonorrhoeae* genomes; 368 (0.04%) were classified as DS. Of the models evaluated, the model trained with a random forest classifier had the highest performance (AUC 0.965; bAcc 0.926). A model retrained the top five SNPs, according to SHAP values, demonstrated a similar performance (AUC 0.916; bAcc 0.879) as the model with 97 SNPs. An ML approach using mutations in *N. gonorrhoeae* can be used to predict S/DS to ceftriaxone. The results highlight a practical application of ML to identify mutations most associated with S/DS to ceftriaxone, which can aid in the development of assays to predict AMR.

**IMPORTANCE:**

Antimicrobial resistance in *Neisseria gonorrhoeae* is an urgent global health issue. The objectives of the study were to use a global collection of 12,936 *N*. *gonorrhoeae* genomes from the PathogenWatch database to evaluate different machine learning models to predict ceftriaxone susceptibility/decreased susceptibility using 97 mutations known to be associated with ceftriaxone resistance. We found the random forest classifier model had the highest performance. The analysis also reported the relative contributions of different mutations within the ML model predictions, allowing for the identification of the mutations with the highest importance for ceftriaxone resistance. A machine learning model retrained with the top five mutations performed similarly to the model using all 97 mutations. These results could aid in the development of molecular tests to detect resistance to ceftriaxone in *N. gonorrhoeae*. Moreover, this approach could be applied to building and evaluating machine learning models for predicting antimicrobial resistance in other pathogens.

## INTRODUCTION

*Neisseria gonorrhoeae*, the bacterial pathogen that causes gonorrhea, is one of the most common sexually transmitted infections, with an estimated 82.4 million new infections globally in 2020 (1). Antimicrobial resistance (AMR) in *N. gonorrhoeae* is increasing and is considered an urgent global health issue (2). Currently, ceftriaxone is the last remaining empiric treatment option for gonorrhea, and ceftriaxone monotherapy is recommended in many settings, including the USA and UK (3, 4).

Next-generation sequencing has revolutionized our understanding of bacterial pathogens, in general, and has advanced our knowledge of *N. gonorrhoeae*, in particular, including the determination of AMR mutations, investigating outbreaks, and surveillance (5–7). The establishment of large databases of genomic sequence data provides a rich resource for public health professionals and researchers seeking to understand trends of *N. gonorrhoeae* on a global scale. One such database is PathogenWatch (https://pathogen.watch/), a publicly available database that combines sequence data with metadata, including phenotypic AMR data, that can be used not only for surveillance purposes but also for the development of molecular assays to improve diagnosis and treatment (8, 9).

Using bacterial genomic data, prior research has used machine learning (ML) algorithms to predict AMR in various pathogens. For example, Nguyen et al. used extreme gradient boosting to predict MIC values for nontyphoidal *Salmonella* species against multiple antibiotics (10). Analyses of different ML models for predicting AMR in *N. gonorrhoeae* to different antibiotics, including ciprofloxacin, cefixime, and azithromycin, found performance varied depending on resistance metrics, antibiotic drug, and ML model, highlighting the complexity of developing clinically applicable ML models (11, 12). Another study employed an artificial intelligence method to identify known and unknown SNPs associated with resistance to penicillin, tetracycline, azithromycin, ciprofloxacin, and cefixime using *N. gonorrhoeae* genomic data (13).

When training an ML model, two types of input data can be used with genomic data, namely, k-mer-based and reference-based (14). A k-mer method has an advantage when the clinical reference is not set and if pathogens have complex AMR mechanisms. On the other hand, the reference-based method incorporates well-established prior knowledge such as certain mutations in AMR genes. *N. gonorrhoeae* has been studied extensively and many mutations are known to be associated with resistance to ceftriaxone (15, 16). Demczuk et al. used multivariate regression to create an equation for predicting minimum inhibitory concentration (MIC) values of *N. gonorrhoeae* using a number of antibiotics, including ceftriaxone, within a data set of Canadian isolates (17). However, machine learning approaches using a global database of genomic data to predict resistance to ceftriaxone have not yet been accomplished.

When training an ML model, two types of input data can be used with genomic data, namely, k-mer-based and reference-based (14). A k-mer method has an advantage when the clinical reference is not set and if pathogens have complex AMR mechanisms. On the other hand, the reference-based method incorporates well-established prior knowledge such as certain mutations in AMR genes. *N. gonorrhoeae* has been studied extensively and many mutations are known to be associated with resistance to ceftriaxone (15, 16). Demczuk et al. used multivariate regression to create an equation for predicting minimum inhibitory concentration (MIC) values of *N. gonorrhoeae* using a number of antibiotics, including ceftriaxone, within a data set of Canadian isolates (17). However, machine learning approaches using a global database of genomic data to predict resistance to ceftriaxone have not yet been accomplished.

The objectives of this study were to use the global PathogenWatch database to develop, evaluate, and compare several different machine learning algorithms that use reference-based genotypic data to predict susceptibility/decreased susceptibility of *N. gonorrhoeae* to ceftriaxone.

(The results in this study were presented at the 11th International Conference on Emerging Infectious Diseases [7 to 10 August 2022] in Atlanta, GA, USA.)

## RESULTS

### Most *N. gonorrhoeae* strains were susceptible to ceftriaxone

**Fig 1:**
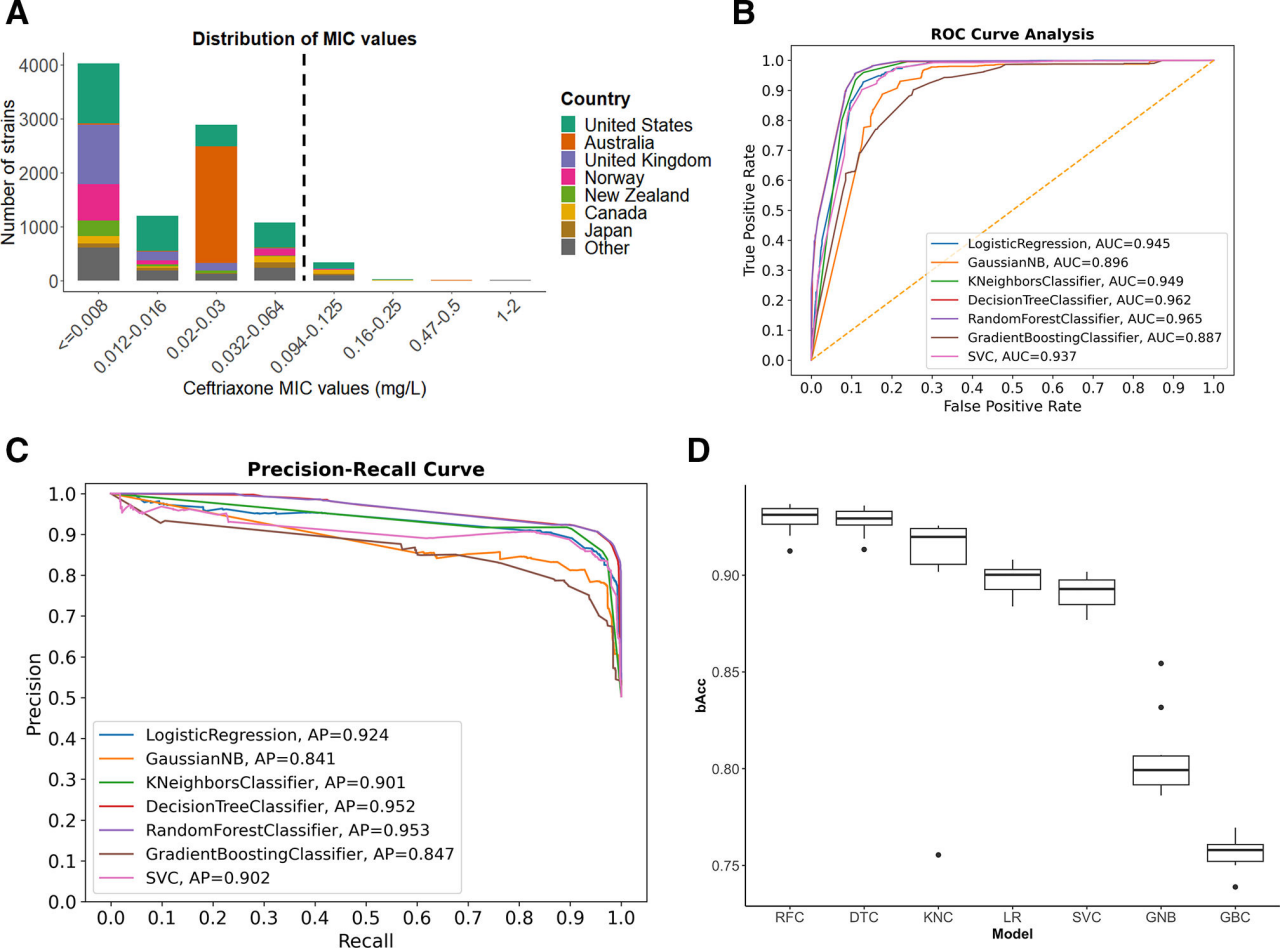
Assessment of different genetic mutations reveals optimal performance by RFC in predicting ceftriaxone susceptibility/decreased-susceptibility phenotypes. (**A**) The MIC value distribution and regional prevalence of *N. gonorrhoeae* isolates are presented, with the dashed line indicating the S (≤0.064 mg/L)/DS (>0.064 mg/L) threshold. A higher proportion of strains from developed nations were present. (**B–D**) Seven distinct machine learning models were developed to predict ceftriaxone susceptibility/decreased susceptibility utilizing 97 SNPs, and their performance metrics were compared. (**B**) Receiver operating characteristic (ROC) curves for each of the models. (**C**) Precision-recall curves for individual models, and (**D**) a box plot demonstrating balanced accuracy derived from 10-fold cross-validation outcomes. Abbreviations: LR, logistic regression; GNB, Gaussian naïve Bayes classifier; KNC, k-nearest neighbors classifier; DTC, decision tree classifier; RFC, random forest classifier; GBC, gradient boosting classifier; SVC, support vector machine.

In total, there were 12,936 genome sequences extracted from PathogenWatch and 9,540 sequences with MIC data included in the machine learning analyses. Among those in the machine learning analyses, most *N. gonorrhoeae* sequences were from the USA and other high-income countries (Fig. 1A; Fig. S1). In total, 368 (0.04%) strains were associated with ceftriaxone MICs > 0.064 mg/L and classified as decreased-susceptible. The low number of strains with decreased susceptibility leads to an imbalance in differentiating outcome classes (susceptible vs decreased-susceptible strains) and introduces bias when training the machine learning models. The synthetic minority oversampling technique (SMOTE) was used to generate additional data for 8,804 decreased-susceptible synthetic sequences, resulting in 18,344 total sequences (9,171 susceptible +368 decreased-susceptible +8,804 decreased-susceptible^SMOTE^) (Fig. S2).

### Comparisons of seven different machine learning models

We evaluated seven different machine learning algorithms on 18,344 real and synthetic *N. gonorrhoeae* sequences. Six different performance metrics were used to evaluate the models, namely, average precision (AP), sensitivity, accuracy, area under the curve (AUC) from the receiver operating characteristic curve, balanced accuracy (bAcc), and F1 score. The AUC, AP, and bAcc scores for each model are depicted in Fig. 1B through D, respectively, while other score measures such as accuracy (0.755–0.926) and F1 score (0.713–0.928) are listed in Table 1. Among the seven machine learning algorithms, the model trained with the random forest classifier algorithm achieved the highest performance, having the top scores in precision (0.953), recall (0.954), accuracy (0.926), F1 score (0.928), AUC (0.965), and bAcc (0.926). Thus, the random forest classifier model was selected for the prediction of ceftriaxone susceptibility/decreased susceptibility and further analysis.

**Fig 2:**
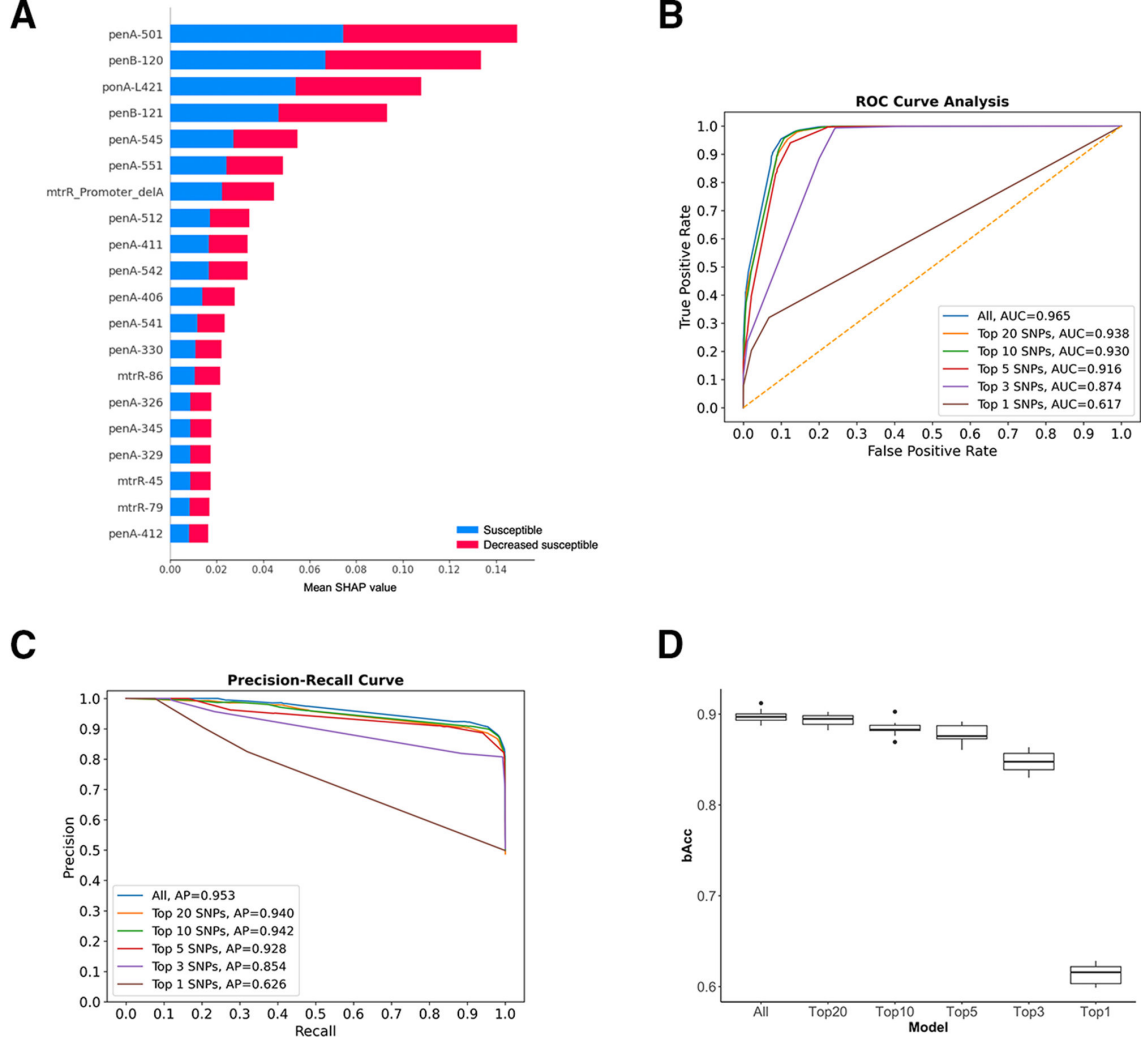
Impact of individual SNPs on RFC model predictions was determined using Shapley additive explanation (SHAP) values, elucidating the significance of individual SNPs within the RFC model. (**A**) The 20 SNPs with the highest SHAP values. Genes and corresponding SNPs are both highlighted. (**B and C**) Receiver operating characteristic (ROC) curves (**B**) and precision-recall curves (**C**) using different combinations of SNPs selected according to their SHAP values. (**D**) A box plot that graphically represents the balanced accuracy of the RFC model. The plot encompasses scenarios where all 97 SNP positions are utilized for training, in addition to the instances where only the top 20, 10, 5, or even just 1 SNP position is employed, chosen based on their SHAP values. It’s noteworthy that even when employing only the top 5 SNPs, the observed variations in model performance remain marginal.

**TABLE 1:** The mean scores of machine-learning models in 10-fold cross-validation^*a*^

Classifier	Precision	Sensitivity	Accuracy	AUC	bAcc	F1 score
LogisticRegression	0.924	0.917	0.9	0.945	0.901	0.902
GaussianNB	0.841	0.976	0.801	0.896	0.802	0.831
KNeighbors	0.901	0.951	0.913	0.949	0.913	0.916
DecisionTree	0.952	0.954	0.926	0.962	0.926	0.928
RandomForest	0.953	0.954	0.926	0.965	0.926	0.928
GradientBoosting	0.847	0.611	0.755	0.887	0.755	0.713
SVC	0.902	0.936	0.891	0.937	0.891	0.896

^*a*^ AUC, area under the curve; bACC, balanced accuracy.

### Mutations impacting the prediction power of random forest classifier

To tease apart the random forest classifier model and the factors associated with predicting susceptibility, the Shapley additive explanation (SHAP) values were calculated for each of the 97 SNPs initially used to train the model in order to measure the impact of each feature in the random forest classifier model (Fig. 2A). The SHAP value originates from the game theory, and it represents the relative contribution of each feature, which, in our study, are the 97 SNPs included in our analysis. Based on the list of SHAP values of individual SNPs, the random forest classifier model was retrained with a smaller set of the top SNPs. Our initial hypothesis was that since each SNP is associated with varying degrees of changes to the involved proteins—for example, folding structure, binding affinity to drug, and transcription—and thus has varying degrees of impact on susceptibility, the model trained with top SNPs from the SHAP analysis should show only a marginal drop of performance. Indeed, when the top 20 SNPs were used, a small decrease in AUC (0.965–0.938), AP (0.953–094), and balanced accuracy (0.926 to 0.882) were observed compared to the model including all 97 SNPs. The models showed only marginal differences even when restricting to only the top 5 SNPs: *penA*-501, *penB*-120, *ponA*-421, *penB*-121, and *penA*-545 (AUC 0.916, AP 0.928, and bACC 0.875) (Fig. 2B through D; Table 2).

**TABLE 2:** The mean scores of different combinations of SNPs on random forest classifier model in the 10-fold cross validation^*a*^

SNPs	Precision	Sensitivity	Accuracy	AUC	bAcc	F1 score
All	0.953	0.954	0.926	0.965	0.926	0.928
Top 20	0.94	0.93	0.885	0.938	0.885	0.889
Top 10	0.942	0.945	0.883	0.93	0.882	0.890
Top 5	0.928	0.894	0.879	0.916	0.879	0.881
Top 3	0.854	0.968	0.842	0.874	0.842	0.859
Top 1	0.626	0.302	0.614	0.617	0.615	0.440

^*a*^ AUC, area under the curve; bACC, balanced accuracy.

## DISCUSSION

Using a large, global genomic database, we applied several ML models to predict decreased susceptibility to ceftriaxone in *N. gonorrhoeae* and found the RFC model performed best, with very high AUC, AP, and bACC values. Furthermore, analyzing feature contributions data identified the SNPs most associated with ceftriaxone resistance and allowed for the identification of more efficient SNP combinations that performed comparably with the models using all 97 SNPs. Identifying ML models to predict decreased susceptibility of ceftriaxone in *N. gonorrhoeae* and the SNPs highly predictive of S/DS within a global database of *N. gonorrhoeae* genomes are important findings that advance our understanding of ceftriaxone resistance. The ML models can be used for enhanced surveillance and aid in the development of molecular assays to predict ceftriaxone resistance in clinical specimens.

The RFC performed best for predicting ceftriaxone resistance compared to the other six models evaluated in this study. One possible reason the RFC performed better than the other models is its ability to handle missing data and maintain accuracy, which are important features given the presence of partial or missing AMR genetic data due to the incompleteness of genome sequences (18). Despite the incompleteness of genomic data, the high bACC and accuracy indicate the robustness of the RFC model. Still, other studies have shown that the performance of different ML algorithms may vary depending on which sequence and drug are targeted for training (14). Thus, while we observed the RFC was the best ML model for ceftriaxone resistance, other models might work better when evaluating other antibiotics or with other data sets. Additional research and evaluation of other ML models using different data sets or with different pathogens will expand our knowledge of ML approaches to predict AMR.

In this study, the SHAP analysis was used to determine the feature contributions of each SNP on ceftriaxone resistance and identified several SNPs with high impact. We also observed that a combination of the top 5 SNPs was very efficient and showed only a slight decrease in performance, maintaining AUC >90% and bACC >85%. Interestingly, the top 20 SNPs in the SHAP analysis were primarily comprised of mutations within the *penA*, *penB*, and promoter region of *mtrR* genes. Many of those mutations are known to be associated with ceftriaxone resistance, provide strong rationale on why they had the highest impact in the model, and have already been incorporated into molecular assays and algorithms, including our own published work (19–21). For example, a mutation at Ala501 in *penA* leads to increased rigidity in the active site region in penicillin-binding protein two which decreases ceftriaxone binding affinity (22). Similarly, the mutations G120 and A121 in *penB*, which encodes one of the *N. gonorrhoeae* porins, generate a pore constriction zone in loop3 that decreases antibiotic influx (23, 24). In addition, while our prior work used the mosaic *penA* for the prediction of S/DS to ceftriaxone, the current analysis did not find a strong impact of the mosaic *penA* among genomes within this database (20, 25). This finding might indicate mosaicism in *penA* is a less important factor for ceftriaxone decreased susceptibility on a global scale or that genomes with *penA* mosaicism and decreased susceptibility to ceftriaxone were less represented within the PathogenWatch database. Lastly, a deletion in the *mtrR* promoter region (−35delA) is known to repress the expression of *mtrR* and is associated with ceftriaxone resistance through increased expression of the MtrCDE efflux pump (16). However, mutations in the coding region of *mtrR* contribute less to resistance. Out of the 3,728 strains with a −35delA *mtrR* mutation in our analysis, 3,258 strains had a coding mutation at amino acid position 105 (H105Y). This mutation reduces *mtrR* binding to the *mtrCDE* promoter region by 12-fold, mainly due to an allosteric interaction involving residue D68, that reduces target recognition leading to up-regulation (26). Other *mtrR* coding mutations in strains with −35delA *mtrR* were also found: 482 with mtrR-T86A, 476 with D79N, 421 with G45D, 44 with A39T, and one strain with R44H; these mutations are likely transferred along with −35delA *mtrR*, given the highly competent nature of *N. gonorrhoeae*. While coding region mutations were identified, it’s important to note that the −35delA mutation in the promoter region will suppress *mtrR* expression, thereby diminishing the impact of the coding region mutations.

Using genetic markers to guide antibiotic therapy, called resistance-guided therapy, is an emerging concept for *N. gonorrhoeae* (27, 28). Understanding how many SNPs, and in which combinations, can predict resistance to ceftriaxone is important, as they can be used to develop molecular assays to predict ceftriaxone resistance. While antimicrobial susceptible testing (AST) is important for determining AMR phenotypes, the process relies on bacterial culturing and has a long turnaround time. Therefore, incorporating genotypic markers into molecular assays can expedite the detection of AMR and have an impact on treatment decisions. Our report demonstrates how ML approaches could be used to identify promising SNPs to incorporate into molecular assays for diagnostic use in the future. However, phenotypic AST remains critical to generating a full antibiotic susceptibility profile, for surveillance, and to advance our understanding of genotypic-phenotypic relationships; thus, we do not envision molecular assays replacing AST entirely.

Although the ML models generated here show promising results, there are some limitations to this study. First, the distribution of isolates and AMR data are not equal, and a large proportion of data are from high-income countries. The model we trained may therefore be overfitted and somewhat region-specific. Second, even though SMOTE was incorporated to overcome the imbalance in S/DS strains in *N. gonorrhoeae*, there may be a bias toward DS strains because, initially, there were only 368 genomes available. Third, we only used data included in the PathogenWatch database and were limited to the availability and quality of data included in that database. For example, some mutations in RNA polymerase (*rpoB* and *rpoD*) have been associated with *penA*-independent resistance to ceftriaxone; however, these mutations were very rare in our PathogenWatch data set, limiting our ability to include them in our analysis (29). The PathogenWatch database is well-maintained and the data quality is understood to be high (8). Moreover, using the PathogenWatch database was an overall strength of this study, as it provided one of the largest global *N. gonorrhoeae* genomic data sets to develop, train, and test our ML models.

In conclusion, our study generated a robust ML model to predict decreased susceptibility to ceftriaxone using global *N. gonorrhoeae* genomic data from PathogenWatch. We used a state-of-the-art ML technique to avoid overfitting the model and measured the relative impact of each mutation in known AMR genes. The results of this study go beyond simple identification of decreased susceptibility genetic mutations and can be used to guide the development of genotypic testing assays that could be incorporated into diagnostic tests or be used for the surveillance of AMR in *N. gonorrhoeae*. Moreover, the ML methods reported here could prove to be a foundational tool that can be applied to predicting AMR within other pathogens of interest. As AMR continues to increase, ML approaches to predict resistance can aid in the surveillance, diagnosis, and treatment of infections.

## MATERIALS AND METHODS

### Data collection and preprocessing

For the machine learning analysis, a total of 12,936 *N*. *gonorrhoeae* genomes and relevant metadata were collected from the PathogenWatch database on 17 November 2020. The 97 genetic mutations that are most associated with ceftriaxone resistance were extracted using an in-house Python script called mutation detector (https://github.com/smha118/mutation_detector) as described previously (9). The metadata for all strains were obtained from the PathogenWatch database including accession number, country information, and MIC values. Ceftriaxone susceptibility and decreased susceptibility were determined based on MIC values of ≤0.064 and >0.064 mg/L, respectively. Any strains without MIC values were excluded from further analysis (*n* = 3,396). Thus, a total of 9,540 strains were used for machine learning training and prediction. Before conducting the machine learning procedure, all of the nucleotides from the promoter region and amino acids were converted into quantifiable integers as defined in extended data Table 1. Similarly, ceftriaxone susceptibility and decreased susceptibility were classified as 0 and 1 to represent susceptible and decreased-susceptible strains, respectively.

### Machine learning (ML) training and prediction

Python (v3.8.12) was used as a primary coding language where Pandas (v1.2.4), NumPy (v1.20.3), Scikit-learn (v0.24.1), and Matplotlib (v3.4.3) were incorporated for data manipulation, matrix processing, ML analysis, and visualization, respectively (30). Since the data were largely imbalanced between susceptible and decreased-susceptible strains, the synthetic minority oversampling technique (SMOTE), a method known to improve the accuracy of models trained compared to the oversampling with replacement method, was used to generate synthetic DS data using the k-nearest neighbor (KNN) method and oversample training/test data (31). The SMOTE generates new instances from the existing minority features (in this case, the mutation profile of decreased-susceptible strains) by (i) calculating the distance between one another from minor feature vectors, (ii) multiplying by a random number between 0 and 1 to the distances, (iii) adding them back to an original feature vector, and (iv) repeating the process until there are matching number of minor features. Following SMOTE, 10-fold cross-validation was performed using the cross_validate function in the Scikit-learn library in Python. The cross-validation step first randomly splits the data set into 10 data blocks. Subsequently, an iterative process of ML modeling is performed 10 times, with one of the blocks used as a test data set and the nine other blocks used as a training data set. The results of 10 iterations are used to calculate the final performance metrics. The 10-fold cross-validation technique allows for more accurate estimations of the area under the curve (AUC) and average precision (AP). The AUC explains the trade-offs between the true positive rate (sensitivity) as a function of the false positive rate (100 specificity), while AP measures the predictive power of the model that accounts for imbalance in the data set (32).

A total of seven ML algorithms were utilized for evaluation, namely, logistic regression (LR), Gaussian naïve Bayes classifier (GNB), k-nearest neighbors classifier (KNC), decision tree classifier (DTC), random forest classifier (RFC), gradient boosting classifier (GBC), and support vector machine (SVC). The performance of each ML model was evaluated based on precision $\left(\frac{TP}{TP+FP}\right)$, recall $\left(\frac{TP}{TP+FN}\right)$, accuracy $\left(\frac{TP+TN}{TP+FP+TN+FN}\right)$, balanced accuracy ( $\frac{Specificity+Sensitivity}{2}$), F1 score $\left(2\times \frac{precision\;\times \;recall}{precision\;+\;recall}\right)$, and the AUC, where TP, TN, FP, and FN are abbreviations for true positive, true negative, false positive, and false negative, respectively. bAcc is a performance metric similar to accuracy but has adjustments that make it perform better with imbalanced data sets.

### Measuring of individual SNP scores in ML training

The individual SNP contributions for the RFC model were measured using Shapley additive explanation (SHAP) values (v0.40.0) (33). SHAP values were calculated using the TreeExplainer and shap_values functions with the RFC model and training data set as input data, respectively. Afterward, the summary_plot function was used to visualize individual SNP contributions using the calculated SHAP values. The top-scored mutations were targeted for further ML modeling to measure whether certain combinations of mutations are sufficient for the identification of ceftriaxone susceptibility. The same performance metrics (precision, recall, accuracy, bAcc, F1 score, and AUC) were used as in the previous section.

**Fig 3:**
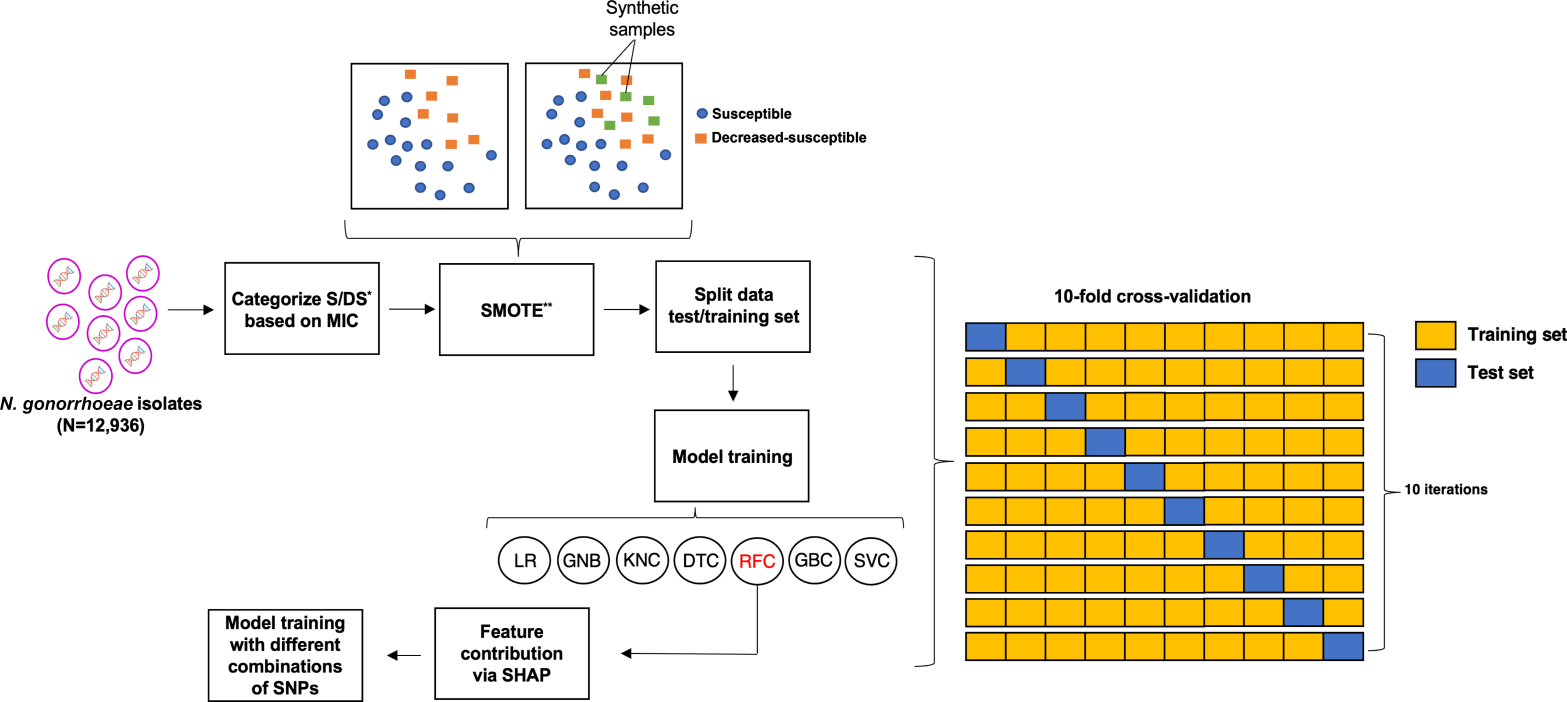
The schematic illustrates the comprehensive workflow adopted in this study. A data set comprising 12,936 *Neisseria gonorrhoeae* genomes sourced from the PathogenWatch database formed the basis of the study. These genomes were subjected to analysis using seven distinct machine learning (ML) models. The random forest classifier emerged as the most adept performer among the various ML models considered. Notably, this classifier was harnessed to compute contribution scores for each distinct mutation under scrutiny. Further enriching the analysis, different combinations of mutations were employed to train additional models. Intriguingly, despite using altered mutation combinations, the resultant models exhibited only marginal decreases in their performance metrics. Abbreviations: LR, logistic regression; GNB, Gaussian naïve Bayes classifier; KNC, k-nearest neighbors classifier; DTC, decision tree classifier; RFC, random forest classifier; GBC, gradient boosting classifier; SVC, support vector machine.

An overview of the study workflow is depicted in Fig. 3.

## Data Availability

All the sequence data we used in this study are available at the PathogenWatch database: https://pathogen.watch/. The code to generate and evaluate the machine learning models is available on GitHub: https://github.com/smha118/ML_evalulation_tool.

## SUPPLEMENTAL MATERIAL

Fig. S1 spectrum.01703-23-s0001.tif Barplot showing the data distribution of *N. gonorrhoeae* strains by different nations.

Fig. S2 spectrum.01703-23-s0002.tif Depiction of how the synthetic minority oversampling technique (SMOTE) generated synthetic ceftriaxone decreased susceptible (DS) strains to balance the data set.

Supplemental legends spectrum.01703-23-s0003.docx Legends for Fig. S1 and S2.

ASM does not own the copyrights to Supplemental Material that may be linked to, or accessed through, an article. The authors have granted ASM a non-exclusive, world-wide license to publish the Supplemental Material files. Please contact the corresponding author directly for reuse.

## References

[1] Unemo M, Lahra MM, Escher M, Eremin S, Cole MJ, Galarza P, Ndowa F, Martin I, Dillon J-A, Galas M, Ramon-Pardo P, Weinstock H, Wi T. 2021. WHO global antimicrobial resistance surveillance for Neisseria gonorrhoeae 2017-18: a retrospective observational study. Lancet Microbe 2:e627–e636. doi:10.1016/S2666-5247(21)00171-3 35544082

[2] Piszczek J, St Jean R, Khaliq Y. 2015. Gonorrhea: treatment update for an increasingly resistant organism. Can Pharm J (Ott) 148:82–89. doi:10.1177/1715163515570111 25918540 PMC4366410

[3] Alirol E, Wi TE, Bala M, Bazzo ML, Chen X-S, Deal C, Dillon J-A, Kularatne R, Heim J, Hooft van Huijsduijnen R, Hook EW, Lahra MM, Lewis DA, Ndowa F, Shafer WM, Tayler L, Workowski K, Unemo M, Balasegaram M. 2017. Multidrug-resistant gonorrhea: a research and development roadmap to discover new medicines. PLoS Med 14:e1002366. doi:10.1371/journal.pmed.1002366 28746372 PMC5528252

[4] Fifer H, Saunders J, Soni S, Sadiq ST, FitzGerald M. 2020. 2018 UK national guideline for the management of infection with Neisseria gonorrhoeae. Int J STD AIDS 31:4–15. doi:10.1177/0956462419886775 31870237

[5] Sánchez-Busó L, Golparian D, Corander J, Grad YH, Ohnishi M, Flemming R, Parkhill J, Bentley SD, Unemo M, Harris SR. 2019. The impact of antimicrobials on gonococcal evolution. Nat Microbiol 4:1941–1950. doi:10.1038/s41564-019-0501-y 31358980 PMC6817357

[6] Grad YH, Goldstein E, Lipsitch M, White PJ. 2016. Improving control of antibiotic-resistant gonorrhea by integrating research agendas across disciplines: key questions arising from mathematical modeling. J Infect Dis 213:883–890. doi:10.1093/infdis/jiv517 26518045 PMC4760416

[7] Buckley C, Forde BM, Trembizki E, Lahra MM, Beatson SA, Whiley DM. 2018. Use of whole genome sequencing to investigate an increase in Neisseria gonorrhoeae infection among women in urban areas of Australia. Sci Rep 8:1503. doi:10.1038/s41598-018-20015-x 29367612 PMC5784116

[8] Sánchez-Busó L, Yeats CA, Taylor B, Goater RJ, Underwood A, Abudahab K, Argimón S, Ma KC, Mortimer TD, Golparian D, Cole MJ, Grad YH, Martin I, Raphael BH, Shafer WM, Town K, Wi T, Harris SR, Unemo M, Aanensen DM. 2021. A community-driven resource for genomic epidemiology and antimicrobial resistance prediction of Neisseria gonorrhoeae at Pathogenwatch. Genome Med 13:61. doi:10.1186/s13073-021-00858-2 33875000 PMC8054416

[9] Adamson PC, Lin EY, Ha S-M, Klausner JD. 2021. Using a public database of Neisseria gonorrhoeae genomes to detect mutations associated with zoliflodacin resistance. J Antimicrob Chemother 76:2847–2849. doi:10.1093/jac/dkab262 34324655 PMC8521401

[10] Nguyen M, Long SW, McDermott PF, Olsen RJ, Olson R, Stevens RL, Tyson GH, Zhao S, Davis JJ. 2019. Using machine learning to predict antimicrobial MICs and associated genomic features for nontyphoidal Salmonella. J Clin Microbiol 57:e01260-18. doi:10.1128/JCM.01260-18 30333126 PMC6355527

[11] Hicks AL, Wheeler N, Sánchez-Busó L, Rakeman JL, Harris SR, Grad YH. 2019. Evaluation of parameters affecting performance and reliability of machine learning-based antibiotic susceptibility testing from whole genome sequencing data. PLoS Comput Biol 15:e1007349. doi:10.1371/journal.pcbi.1007349 31479500 PMC6743791

[12] Yasir M, Karim AM, Malik SK, Bajaffer AA, Azhar EI. 2022. Prediction of antimicrobial minimal inhibitory concentrations for Neisseria gonorrhoeae using machine learning models. Saudi J Biol Sci 29:3687–3693. doi:10.1016/j.sjbs.2022.02.047 35844400 PMC9280306

[13] Shi J, Yan Y, Links MG, Li L, Dillon J-A, Horsch M, Kusalik A. 2019. Antimicrobial resistance genetic factor identification from whole-genome sequence data using deep feature selection. BMC Bioinformatics 20:535. doi:10.1186/s12859-019-3054-4 31874612 PMC6929425

[14] Anahtar MN, Yang JH, Kanjilal S. 2021. Applications of machine learning to the problem of antimicrobial resistance: an emerging model for translational research. J Clin Microbiol 59:e0126020. doi:10.1128/JCM.01260-20 33536291 PMC8218744

[15] Unemo M, Seifert HS, Hook EW, Hawkes S, Ndowa F, Dillon J-AR. 2019. Gonorrhoea. Nat Rev Dis Primers 5:79. doi:10.1038/s41572-019-0128-6 31754194

[16] Unemo M, Shafer WM. 2014. Antimicrobial resistance in Neisseria gonorrhoeae in the 21st century: past, evolution, and future. Clin Microbiol Rev 27:587–613. doi:10.1128/CMR.00010-14 24982323 PMC4135894

[17] Demczuk W, Martin I, Sawatzky P, Allen V, Lefebvre B, Hoang L, Naidu P, Minion J, VanCaeseele P, Haldane D, Eyre DW, Mulvey MR. 2020. Equations to predict antimicrobial MICs in Neisseria gonorrhoeae using molecular antimicrobial resistance determinants. Antimicrob Agents Chemother 64:e02005-19. doi:10.1128/AAC.02005-19 31871081 PMC7038236

[18] Petrazzini BO, Naya H, Lopez-Bello F, Vazquez G, Spangenberg L. 2021. Evaluation of different approaches for missing data imputation on features associated to genomic data. BioData Min 14:44. doi:10.1186/s13040-021-00274-7 34479616 PMC8414708

[19] Lin EY, Adamson PC, Deng X, Klausner JD. 2021. Establishing novel molecular algorithms to predict decreased susceptibility to ceftriaxone in Neisseria gonorrhoeae strains. J Infect Dis 223:1232–1240. doi:10.1093/infdis/jiaa495 32779717 PMC8030720

[20] Lin EY, Adamson PC, Ha S-M, Klausner JD. 2022. Reliability of genetic alterations in predicting ceftriaxone resistance in Neisseria gonorrhoeae globally. Microbiol Spectr 10:e0206521. doi:10.1128/spectrum.02065-21 35348352 PMC9045316

[21] Peterson SW, Martin I, Demczuk W, Bharat A, Hoang L, Wylie J, Allen V, Lefebvre B, Tyrrell G, Horsman G, Haldane D, Garceau R, Wong T, Mulvey MR. 2015. Molecular assay for detection of genetic markers associated with decreased susceptibility to cephalosporins in Neisseria gonorrhoeae. J Clin Microbiol 53:2042–2048. doi:10.1128/JCM.00493-15 25878350 PMC4473228

[22] Tomberg J, Fedarovich A, Vincent LR, Jerse AE, Unemo M, Davies C, Nicholas RA. 2017. Alanine 501 mutations in penicillin-binding protein 2 from Neisseria gonorrhoeae: structure, mechanism, and effects on cephalosporin resistance and biological fitness. Biochemistry 56:1140–1150. doi:10.1021/acs.biochem.6b01030 28145684 PMC5502787

[23] Olesky M, Zhao S, Rosenberg RL, Nicholas RA. 2006. Porin-mediated antibiotic resistance in Neisseria gonorrhoeae: ion, solute, and antibiotic permeation through PIB proteins with penB mutations. J Bacteriol 188:2300–2308. doi:10.1128/JB.188.7.2300-2308.2006 16547016 PMC1428387

[24] Lee S-G, Lee H, Jeong SH, Yong D, Chung GT, Lee YS, Chong Y, Lee K. 2010. Various penA mutations together with mtrR, porB and ponA mutations in Neisseria gonorrhoeae isolates with reduced susceptibility to cefixime or ceftriaxone. J Antimicrob Chemother 65:669–675. doi:10.1093/jac/dkp505 20093260 PMC2837549

[25] Lin EY, Adamson PC, Klausner JD. 2022. Applying molecular algorithms to predict decreased susceptibility to ceftriaxone from a report of strains of Neisseria gonorrhoeae in Amsterdam, the Netherlands. J Antimicrob Chemother 77:534–536. doi:10.1093/jac/dkab389 34741618 PMC8809185

[26] Beggs GA, Ayala JC, Kavanaugh LG, Read TD, Hooks GM, Schumacher MA, Shafer WM, Brennan RG. 2021. Structures of Neisseria gonorrhoeae MtrR-operator complexes reveal molecular mechanisms of DNA recognition and antibiotic resistance-conferring clinical mutations. Nucleic Acids Res 49:4155–4170. doi:10.1093/nar/gkab213 33784401 PMC8053128

[27] Klausner JD, Bristow CC, Soge OO, Shahkolahi A, Waymer T, Bolan RK, Philip SS, Asbel LE, Taylor SN, Mena LA, Goldstein DA, Powell JA, Wierzbicki MR, Morris SR. 2021. Resistance-guided treatment of gonorrhea: a prospective clinical study. Clin Infect Dis 73:298–303. doi:10.1093/cid/ciaa596 32766725 PMC8282307

[28] Allan-Blitz L-T, Adamson PC, Klausner JD. 2022. Resistance-guided therapy for Neisseria gonorrhoeae. Clin Infect Dis 75:1655–1660. doi:10.1093/cid/ciac371 35818315 PMC10200317

[29] Palace SG, Wang Y, Rubin DH, Welsh MA, Mortimer TD, Cole K, Eyre DW, Walker S, Grad YH. 2020. RNA polymerase mutations cause cephalosporin resistance in clinical Neisseria gonorrhoeae isolates. Elife 9:e51407. doi:10.7554/eLife.51407 32011233 PMC7012608

[30] Varoquaux F, Varoquaux G, Gramfort A, Michel V, Thirion B, Grisel O, Blondel M, Prettenhofer P, Weiss R, Dubourg V, Vanderplas J, Passos A, Cournapeau D, Brucher M, Perrot M, Duchesnay E. 2011. Scikit-learn: machine learning in Python. J Mach Learn Res:2825–2830. doi:10.1007/978-3-642-22092-0

[31] Chawla NV, Bowyer KW, Hall LO, Kegelmeyer WP. 2002. SMOTE: synthetic minority over-sampling technique. J Artif Intell Res 16:321–357. doi:10.1613/jair.953

[32] Yuan Y, Su W, Zhu M. 2015. Threshold-free measures for assessing the performance of medical screening tests. Front Public Health 3:57. doi:10.3389/fpubh.2015.00057 25941668 PMC4403252

[33] Lundberg SM, Lee SI. 2017. A unified approach to interpreting model predictions. Adv Neur In 30:4765–4774.

