# Supplementary figures and images for: Machine learning to predict ceftriaxone resistance using single nucleotide polymorphisms within a global database of Neisseria gonorrhoeae genomes
Source: Microbiol Spectr. 2023 Oct 31;11(6):e01703-23. doi: 10.1128/spectrum.01703-23 (PMC10714741; doi:10.1128/spectrum.01703-23)

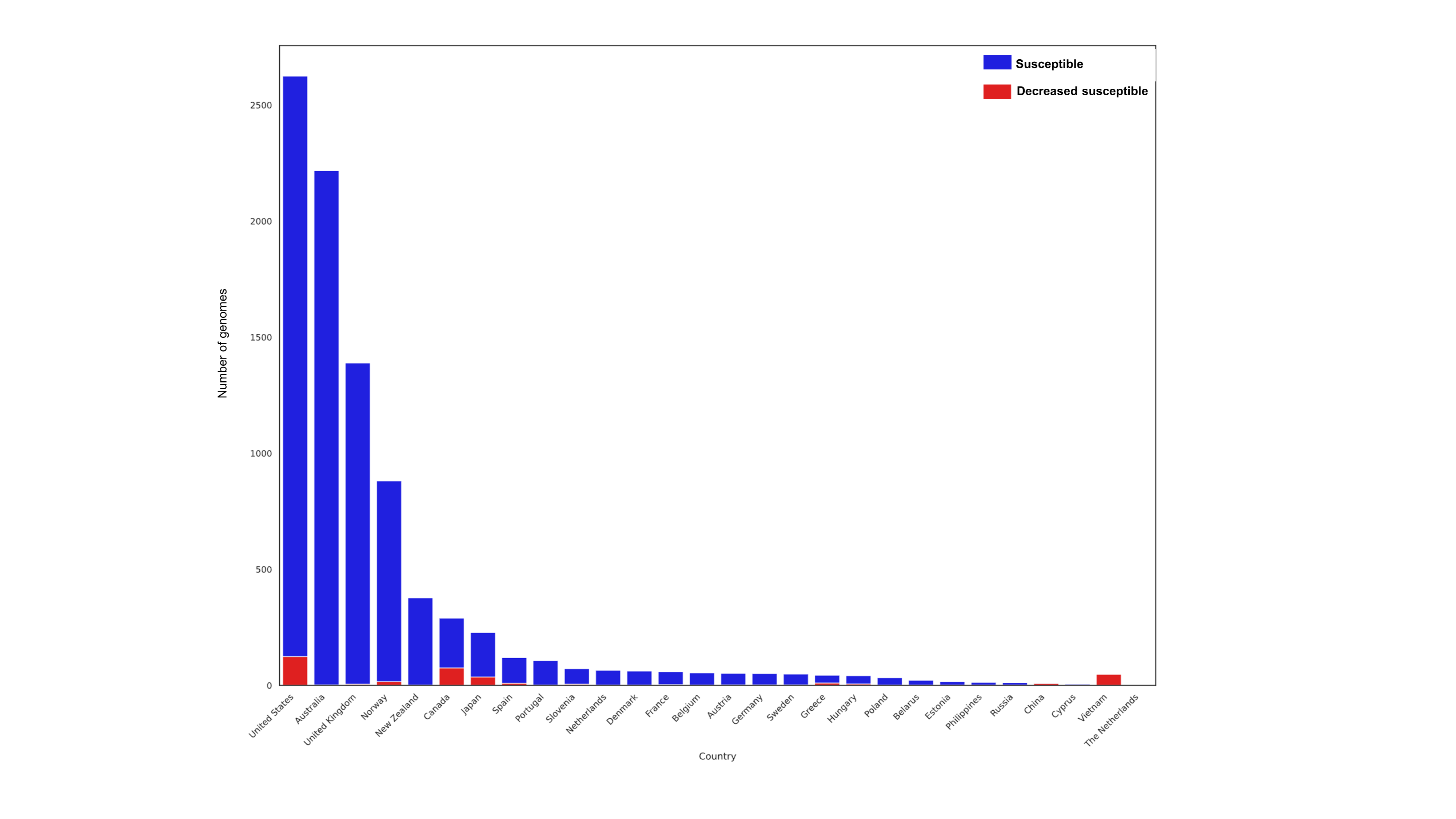

Supplement: Fig. S1 — Barplot showing the data distribution of N. gonorrhoeae strains by different nations. [file spectrum.01703-23-s0001.tif]

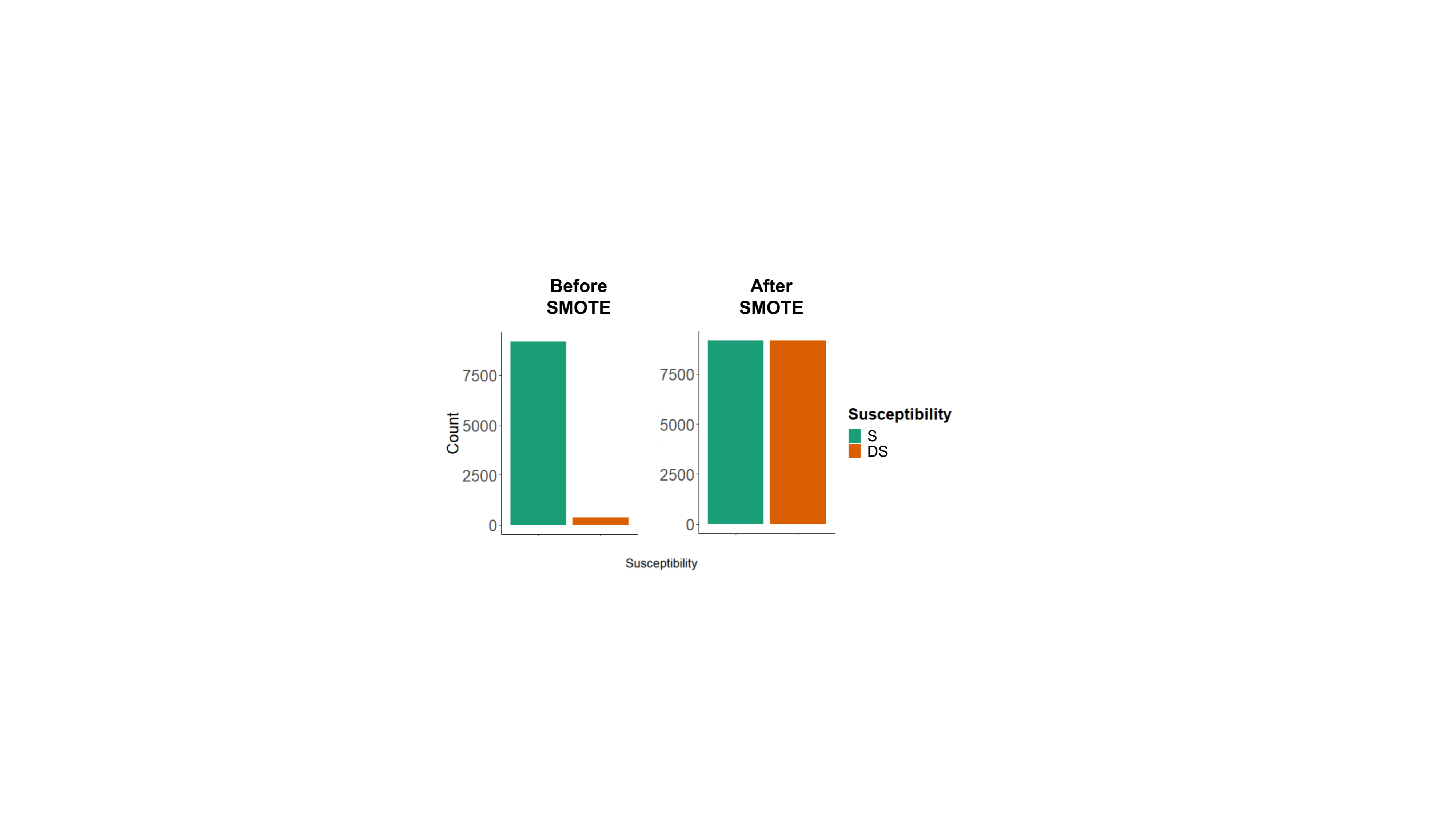

Supplement: Fig. S2 — Depiction of how the synthetic minority oversampling technique (SMOTE) generated synthetic ceftriaxone decreased susceptible (DS) strains to balance the data set. [file spectrum.01703-23-s0002.tif]
