# Supplementary material for: Machine learning to predict ceftriaxone resistance using single nucleotide polymorphisms within a global database of Neisseria gonorrhoeae genomes
Source: Microbiol Spectr. 2023 Oct 31;11(6):e01703-23. doi: 10.1128/spectrum.01703-23 (PMC10714741; doi:10.1128/spectrum.01703-23)
Supplement: Supplemental legends — Legends for Fig. S1 and S2. [file spectrum.01703-23-s0003.docx]

**Supplementary figure legends**

Figure S1. Barplot showing the data distribution of *N. gonnorrhoeae* strains by different nations.

Figure S2. Depiction of how the synthetic minority oversampling technique (SMOTE) generated synthetic DS strains to balance the dataset.
